# MRI findings of epipericardial fat necrosis: As a rare cause of acute chest pain in a healthy man

**DOI:** 10.1016/j.radcr.2022.04.013

**Published:** 2022-05-09

**Authors:** Moezedin Javad Rafiee, Mahya Khaki, Leila Haririsanati, Faranak Babaki Fard, Michael Chetrit, Matthias G. Friedrich

**Affiliations:** a Department of medicine and radiology, McGill University Health Centre, 1001 boul. Decarie, Montreal QC H4A3J1, Montreal, Québec, Canada; b Research Institute, McGill University Health Centre, 1001 boul. Decarie, Montreal QC H4A3J1, Montreal, Québec, Canada; c Faculty of Medicine, University of Montreal, 2900 Boulevard Édouard-Montpetit, Montréal QC H3T 1J4, Montreal, Québec, Canada; d Department of medicine, Division of cardiology, McGill University Health Centre, Montreal, Québec, Canada, 1001 boul. Decarie, Montreal QC H4A3J1; e Department of medicine and radiology, McGill University Health Centre, 1001 boul. Decarie, Montreal QC H4A3J1, Montreal, Québec, Canada

**Keywords:** Magnetic resonance imaging (MRI), Computed tomography (CT), Chest pain, Pericardium, Fat necrosis

## Abstract

Epipericardial fat necrosis (EPFN) is a rare, benign cause of acute chest pain imitating symptoms of life-threatening diseases, such as acute coronary syndrome. Here We report a 37-year-old, healthy male presented to the emergency department (ED) with sudden-onset pleuritic chest pain after an isometric physical training. Initial cardiac workup included ECG, echocardiography was unremarkable, but diagnosis of an inflammatory process that involved the epipericardial fat tissue surrounding the heart was made by showing encapsulated fatty lesion, enhanced adjacent parietal pericardium using of contrast‐enhanced magnetic resonance imaging (MRI). Magnetic resonance imaging would help physicians to differentiate EPFN from severe and life-treating conditions.

## Introduction

Fat necrosis can occur at multiple sites in the body: in the breast and subcutaneous fat, in the abdomen, and in the mediastinum. The etiology of fat necrosis is various as trauma, torsion, and inflammation.

Epipericardial fat necrosis is an inflammatory process that involves pericardial and adjacent mediastinal fat and leads to acute chest pain [1,2]. This is an uncommon, benign, and self-limiting condition that was first described in 1957 [3].

CT scan has been suggested as a modality of choice to detect EPFN [1,3] which demonstrated EPFN as an encapsulated fatty lesion with thickening adjacent pericardium. In the English- language medical literature, the MRI findings of pericardial fat necrosis have been reported in only few case reports [1,6].

## Case presentation

A 37-year-old man presented to the emergency department (ED) with a sudden-onset pleuritic pain in his right anterior lower chest radiating to the upper abdomen. The symptoms started a day before to presentation, shortly after a powerlifting training session. The pain was graded 6 of 10 on a 1-0 scale by patient. There were no associated signs or symptoms such as fever, chills, palpitations, syncopes, cough, or shortness of breath. His physical exam was unremarkable, with a normal resting blood pressure, normal heart sound without murmurs or friction rub, and normal breath sounds with no wheezing or crackles. The patient did not report any significant past medical history and cardiovascular risk factors. An electrocardiogram (ECG) showed normal sinus rhythm with no ST, T wave changes. The laboratory tests, including the D-dimer: 100 ng/L (Reference range <500 ng/L), troponin I: <0.01 (Reference range < 0.016 ng/L), CRP: 2.0 mg/mL (Reference range < 8.0 mg/l) were normal. A transthoracic echocardiography (TTE) reportedly showed normal global systolic left ventricular function without regional wall motion abnormality. There was no evidence of significant pericardial effusion.

A broad range of causes from life-threatening aetiologies to self-limited ones should be considered in the differential diagnosis of acute chest pain. It was including myocardial ischemia, pulmonary embolism, myocarditis, pericarditis, pneumonia, musculoskeletal etiologies, and upper abdominal pathologies such as acute cholecystitis and hepatitis [1,2].

**Fig. 1:**
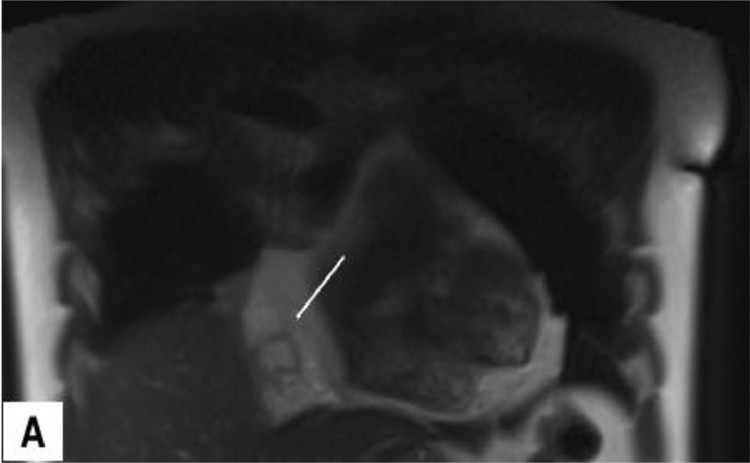
T2-W image in coronal plane: Well encapsulated, oval shape high signal intensity lesion:18 x 11 mm with thin peripheral hypointense rim (white arrow) in the right pericardial fat.

**Fig. 2:**
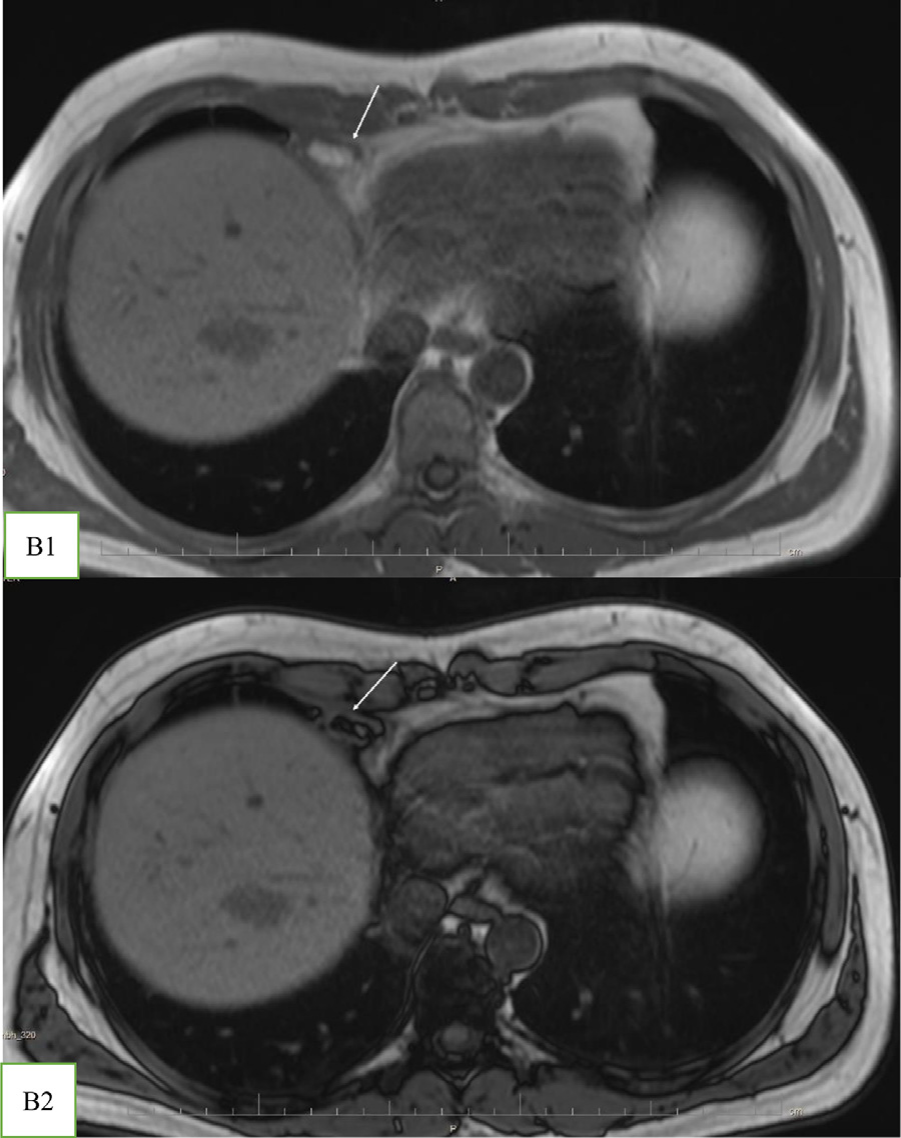
(A) T1 In-phase and (B) Out -of- phase images, as a sensitive technique for confirming fat by showing significant signal loss in the lesion on the out-of-phase sequence.

**Fig. 3:**
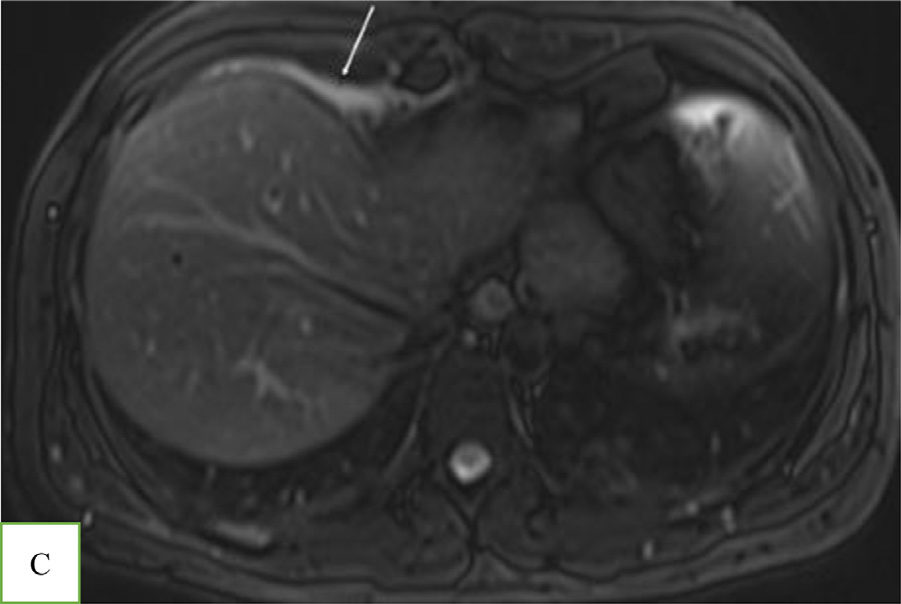
T2-W axial image demonstrates: Small right pleural effusion.

**Fig. 4:**
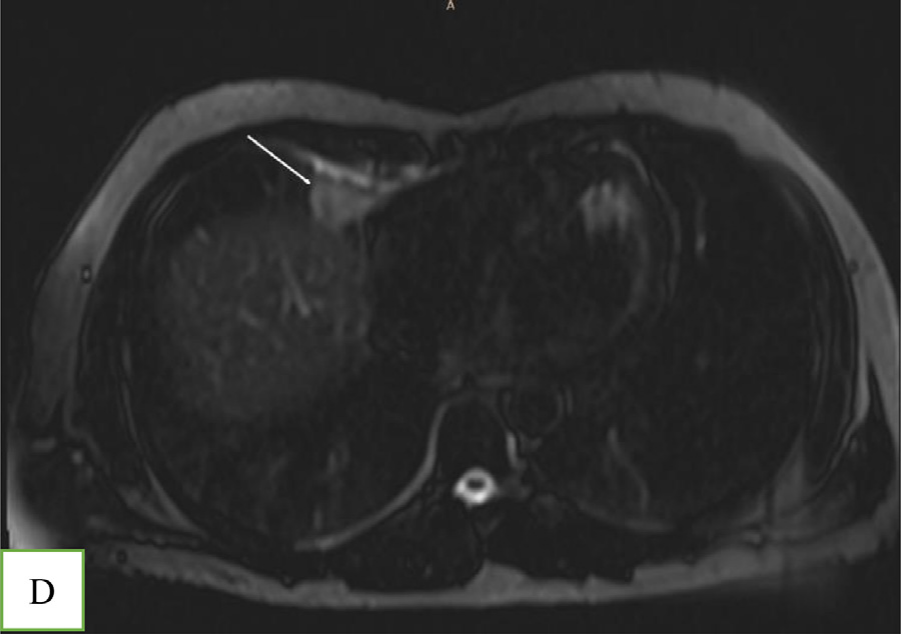
T2-W fat suppressed at level of cardiophrenic angle showing: Subsegmental atelectasis of adjacent right middle lobe .

**Fig. 5:**
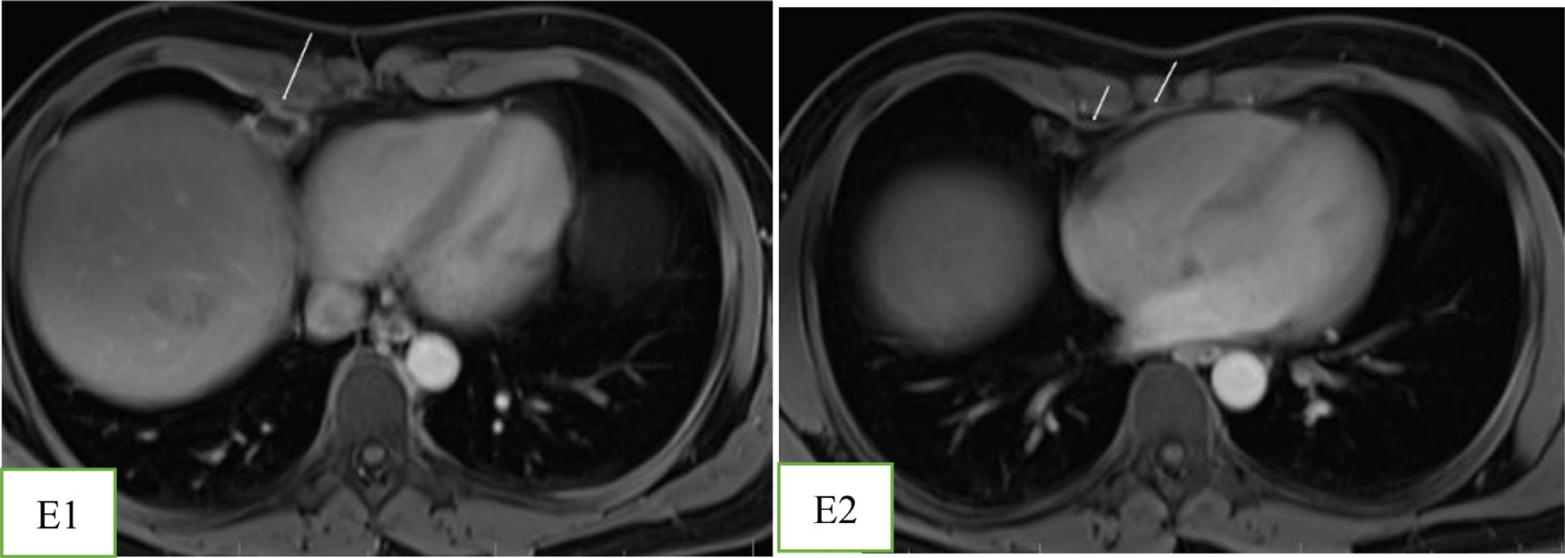
(A) – and (B) T1- W with contrast in axial plane at the level of right cardiophrenic space showing: Enhancement of the peripheral rim of lesion (short arrow). There is a smooth and linear enhancement of the adjacent inflamed pericardium and pleura (arrows).

With the suspicion of muscle strain, the emergency physician prescribed analgesic medication for the patient. Due to the poor response to treatment and the radiating nature of the chest pain to the upper abdomen, he was referred for a contrast-enhanced MRI. A 1.5 T MRI revealed an encapsulated, oval-shaped, and fat-containing nodular lesion (18 × 11 mm) with a surrounding thin, low signal intensity rim, that was located in the right cardiophrenic angle (Figs. 1 and 2). A small reactive pleural effusion with mild, focal thickening of adjacent pleura (Fig. 3) and pericardium in the right cardiophrenic space was observed. Also, subsegmental atelectasis of the right middle lobe was evident (Fig. 4). In the contrast phase study, linear enhancement of the adjacent inflamed pleura and pericardium was noted (Figs. 5A and B). These findings were suggestive for a focal inflammatory etiology.

Based on the patient history, laboratory test results, clinical and imaging findings, diagnosis of EPFN was made. The course of disease was benign and self-limited. On follow-up evaluations at 1, 3, and 6 months: the patient's pain was completely disappeared.

Radiologic follow-up was not performed.

## Discussion

EPFN is a rare and self-limited cause of acute chest pain, mimicking life-threatening diseases like myocardial infarction and pulmonary embolism [2]. Its prevalence in the general population is unknown; however, a review of CT scans in 3604 patients presenting to ED with chest pain reported a frequency of 2.58% [1].

EPFN affects both genders equally, with an average age of 50 [2]. Its underlying etiologies remain unclear [3]; obesity has been suggested as a risk factor [2]. Previous studies proposed some potential mechanisms, including acute torsion of the pericardial fat appendage, hemorrhagic necrosis due to increased intravascular pressure during Valsalva maneuver, and trauma to structural abnormalities, such as lipoma and hamartoma by the beating heart or diaphragm movement [3]. In the presented case, performing Valsalva maneuver during powerlifting may have played a role in the pathogenesis.

EPFN frequently involves the left side [3] and is characterized by a sudden onset pleuritic chest pain lasting between a few hours to several days [1]. Uncommon presentations include dyspnea, tachycardia, and diaphoresis [1]. ECG, laboratory tests, and physical examinations are usually unremarkable [4]. Given the benign self-limiting nature of this disease, conservative treatment is often recommended [4]. Although surgical excision of encapsulated fat necrosis has been described [10], it seems to be the treatment of choice in only those patients with no self-limited or persistent severe chest pain despite appropriate medication and persistent inflammatory changes in radiology follow-up.

The absence of specific signs and symptoms on history and physical exam besides normal laboratory and ECG findings necessitates the utility of imaging modalities to confirm EPFN.

CT scan has been suggested as a modality of choice to detect EPFN [1,3]. Pineda et al. described the EPFN triad based on CT and clinical findings: acute chest pain, encapsulated fatty lesion with dense strands, and thickening of the pericardium [3]. CT scan, however, has a high risk of exposure to ionizing radiation as well as a limited ability to characterize minimal focal changes in pericardial fat, leading to underdiagnosis of EPFN [1,^5^,6].

MRI with fat-suppression techniques is an accurate method to characterize adipose tissue with a high soft-tissue contrast [2,6]. It can distinguish this pathology from other fat-containing tumors in the anterior mediastinum, such as lipomas and liposarcomas [6]. Cardiac MRI has been approved as a robust and non–invasive imaging modality for the evaluation of acute chest pain [7]. Besides the ability of cardiac MRI to rule out serious etiologies such as myocardial ischemia and myocarditis through assessment of myocardial function, morphology, and tissue characterization, it can detect uncommon causes of chest pain [7]. However, this technique has remained underused for the assessment of chest pain despite recent technical advancements [7]; Only 5 out of 53 cases have used MRI to confirm EPFN in adult patients [2,^4^,^6^,^8^,9]. To the best of our knowledge, our patient is the first case that MRI has demonstrated inflammatory changes in adjacent cardiophrenic space (subsegmental pulmonary opacity, thickening and enhancement of pleura, and small pleural effusion) (Figs. 2,3,4, and 5).

## Conclusion

Pineda et al reported using a combination of clinical and CT scan-based criteria for diagnosis of EPFN [3]. Similarly, we suggest MRI diagnostic criteria for EPFN based on the reported MRI findings [2,^4^,^6^,^8^,9]: 1) acute pleuritic chest pain, 2) an encapsulated fat signal intensity lesion, and 3) Enhancement of adjacent pleura or pericardium. We recommend more performing cardiac MRI as a single-scan assessment to differentiate both life-threatening and self-limited causes of acute chest pain.

## Learning objectives

- To review the clinical and imaging features of EPFN as a rare and underdiagnosed cause of acute chest pain.
- To point out the ability of MRI in the differentiation of EPFN from fat-containing tumors, which reduces the need for complementary invasive and non–invasive diagnostic techniques.
- To highlight the advantages of MRI compared with CT scan in the diagnostic workup of patients with acute chest pain presentation.

## Patient consent

The authors of this article confirm that informed consent for publication of this case report was obtained from the patients.
